# A Five-Year Retrospective Audit on Bone Protection Prescribing in Patients With Fragility Fractures in Primary Care

**DOI:** 10.7759/cureus.45532

**Published:** 2023-09-19

**Authors:** Ishtar A Redman, Vicnesan Sivanesan

**Affiliations:** 1 General Practice, Ealing Hospital, London North West University Healthcare NHS Trust, London, GBR; 2 General Practice, The Mansell Road Practice, London, GBR

**Keywords:** bone protection, primary care audit, osteoporotic fractures, fracture liaison service, fragility fractures

## Abstract

Background

Fragility fractures typically occur in the elderly population due to low-energy trauma in the context of underlying osteoporotic bone disease. These fractures are becoming increasingly more common as the population of the United Kingdom ages, representing a significant public health issue. In the community, a joint care approach is adopted between general practitioners and fracture liaison services for the management of patients with fragility fractures. Despite this, preventive care for these patients remains substandard. This project aimed to conduct an audit of patients with a coded diagnosis of a fragility fracture in our primary care practice to ascertain fracture type and the prescription of bone protective agents. When necessary, the appropriate therapy was commenced per best practice guidelines.

Methodology

A search of patients with the diagnosis of *Fragility fracture* on our electronic patient database, SystmOne, was conducted for the period of April 2019 to April 2023 inclusive. A retrospective audit of electronic patient records was done to identify patient demographic data, fracture types and dates, osteoporosis prescriptions, vitamin D/calcium supplementation, and bone densitometry scan results (dual-energy X-ray absorptiometry).

Results

A total of 47 patients were identified with a coded diagnosis of a *Fragility fracture*, of whom 36 were females and 11 were males. The average age of the patients was 76.89 years with a range of 50 to 97. In total, 49 fractures were identified. More than two-thirds of the fractures identified were either distal forearm or neck of femur fractures (18 and 15, respectively). Of the 47 patients identified, 33 were on bone protection agents. Further, 26 received both bisphosphonates and calcium/vitamin D supplementation. Seven patients were on bisphosphonate monotherapy, and the remaining two patients were on vitamin D/calcium supplementation alone. Of the 47 patients, 12 had neither form of therapy prescribed.

Conclusions

Despite the joint effort between fracture liaison services and general practitioners, the secondary prevention of fragility fractures within the community remains inadequate. Fragility fractures are associated with significant morbidity, mortality, and re-fracture rates and incur significant costs to the National Health Service. Local practitioners must routinely evaluate their data to identify opportunities to improve patient care. Effective and timely treatment could be key to the prevention of new or second fractures.

## Introduction

Fragility fractures, defined as those that have resulted from a low-energy trauma, such as a fall from standing height or less, are often a sign of underlying osteoporosis. It is well established that patients who have sustained one fragility fracture are at significantly increased risk of subsequent fractures, especially in the first two years following their initial fracture [1]. In the United Kingdom (UK), there are an estimated 500,000 fragility fractures annually [2], representing a significant public health issue. Furthermore, the current socioeconomic burden of fragility fractures is enormous, with the hospital costs of hip fractures alone estimated at £1.1 billion [3].

Commencing effective treatment and preventive strategies could be key to the prevention of these secondary fractures. In the UK, the outpatient care of patients with fragility fractures falls within the remit of a joint care agreement between fracture liaison services and general practitioners. Within this model, eligible patients, aged 50 and older within a local community or catchment area, are systematically identified, treated, or referred to relevant specialists by either the fracture liaison team or by their general practitioner.

Current treatment guidelines and standards have been developed to reflect national guidance produced by the National Institute of Health and Care Excellence (NICE), the Scottish Intercollegiate Guidelines Network (SIGN), and the British Geriatric Society. For the purposes of this study, NICE guidelines [4] were used for the derivation of audit standards.

## Materials and methods

The primary aim of this audit was to assess the prescription of bone protection chemoprophylaxis in patients with fragility fractures in the community. Secondary outcome measures included the prevalence and types of fragility fractures, in addition to the prescription of bone protection adjuncts (vitamin D and calcium supplementation).

A retrospective audit of all patients with a coded diagnosis of a fragility fracture at Mansell Road Practice in London, from April 2019 to April 2023 inclusive, was conducted. The inclusion criterion for the preliminary search was a coded diagnosis of a fragility fracture. A search of the electronic health records of all patients registered at the practice within this period was done.

Patient electronic records, discharge letters, operation notes, and clinic letters were screened to identify demographic data, fracture types and dates, osteoporosis prescriptions (bisphosphonates, biological therapies, parathyroid analogs, selective estrogen receptor modulators, hormone replacement therapy), vitamin D/calcium supplementation and bone densitometry scan results (dual-energy X-ray absorptiometry, DEXA).

Fractures were grouped into the following categories for the purposes of data collection: distal forearm (radius/ulna), neck of femur, vertebral body, proximal humerus, elbow, lower limb (ankle/tibia/fibula), peri-prosthetic, and other. The data were compiled into an anonymized electronic spreadsheet. Patients with fractures secondary to high-energy mechanisms were excluded from the dataset as these fractures did not satisfy the definition of a fragility fracture (n = 2). Any discrepancies were discussed and resolved between the two authors. The records of these patients were revised and the diagnosis of fragility fracture was removed. An educational poster (Appendices) was created and distributed to the relevant stakeholders within the practice to improve the recognition and electronic coding of fragility fractures.

An in-depth analysis of patients who were not prescribed bone protection agents/calcium/vitamin D supplementation following fragility fractures was conducted to ascertain potential reasons. Guidelines from the NICE were used to supplement clinical decision making and patients were subsequently commenced on treatment, referred for further investigations (DEXA), or advice from specialists sought.

This was a retrospective, single-center, standards-based audit, comparing the practice’s outcomes against national standards. Consequently, the study did not require formal ethical approval per the NHS Health Research Authority’s online decision tool.

## Results

**Figure 1:**
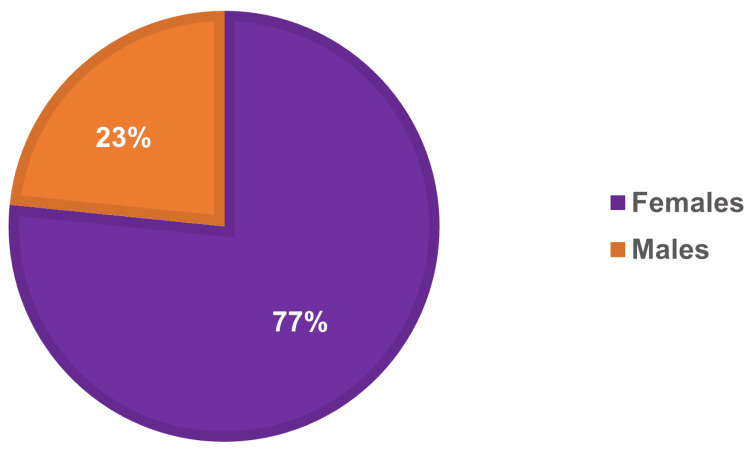
Percentage of male and female patients with fragility fractures at the practice from April 2019 to April 2023.

**Figure 2:**
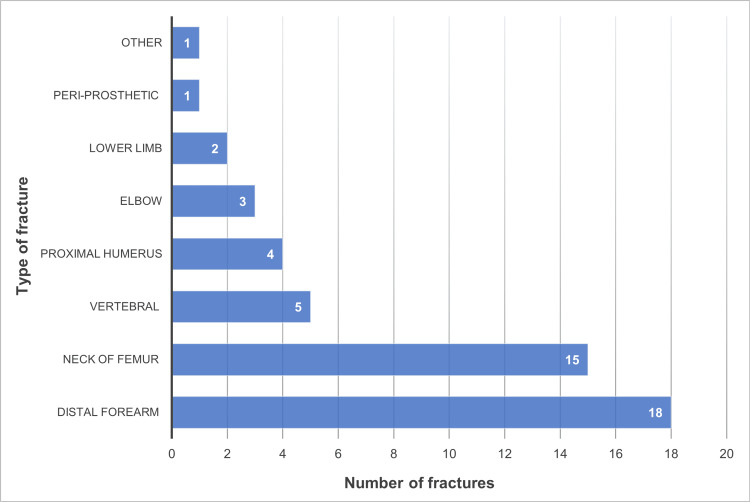
Prevalence of fracture types identified during the study period.

A total of 47 patients were identified with a coded diagnosis of a *Fragility fracture*, of whom there were 36 females and 11 males, a ratio of 3.2:1 (Figure 1). The average age of the patients was 76.89 years (range = 50 to 97). A total of 49 fractures were identified using the above classification system (Figure 2). More than two-thirds of the fractures identified were either distal forearm or neck of femur (18 and 15, respectively).

**Figure 3:**
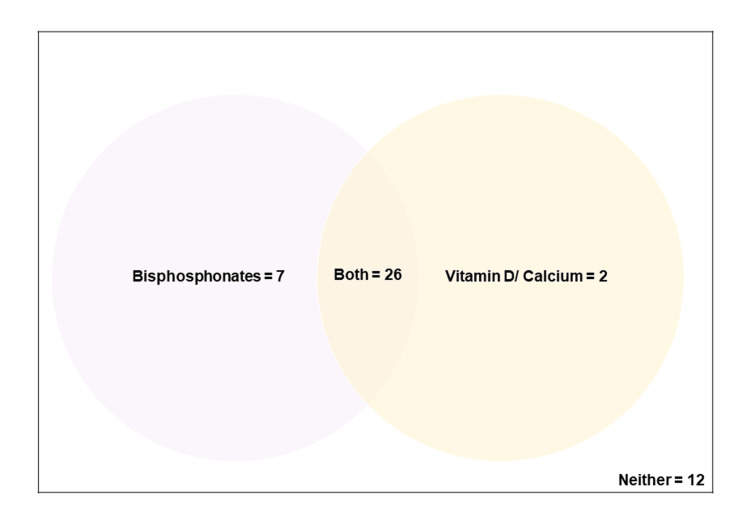
Types of pharmacotherapies prescribed for patients with fragility fractures at the practice during the study period.

Of the 47 patients identified, 33 were on bone protection agents, with 26 receiving both bisphosphonates and calcium/vitamin D supplementation. Seven patients were on bisphosphonate monotherapy and the remaining two patients were on vitamin D/calcium supplementation alone. Of the 47 patients, 12 had neither form of therapy prescribed (Figure 3).

**Figure 4:**
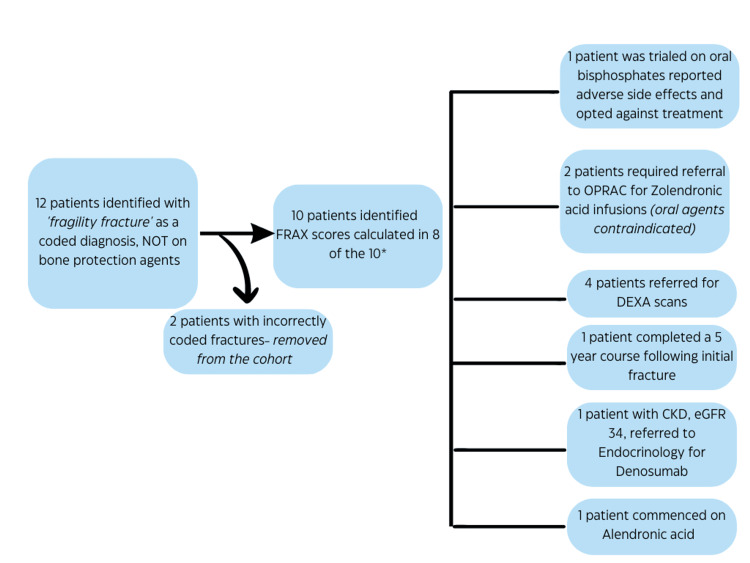
Overview of the results of the search strategy and outcome of the audit. FRAX: Fracture Risk Assessment Tool; DEXA: dual-energy X-ray absorptiometry; CKD: chronic kidney disease; eGFR: estimated glomerular filtration rate; OPRAC: Older Person’s Rapid Access Clinic

Patients who were on bisphosphonate monotherapy were vitamin D and calcium replete. The remaining two patients who were only on vitamin D/calcium supplementation both had recent DEXA scans and Fracture Risk Assessment Tool (FRAX) score calculations which precluded them from needing bone protection agents. An in-depth analysis of the records of the 12 patients on neither therapeutic agent was undertaken to ascertain the reasons for this. A comprehensive breakdown of the remaining 10 patients can be found in Figure 4.

Two of the 12 patients were incorrectly coded for as having a fragility fracture and subsequently removed from the dataset. Of the 10 remaining patients, one was trialed on a bisphosphonate and reported adverse effects, electing against the trial of another class of medication. Four of the 10 patients underwent a bone densitometry scan following their initial fracture. FRAX scores were calculated for the remaining patients and used to guide decision-making regarding the most appropriate drug therapy (Appendices).

## Discussion

The UK has officially entered the era of an aging population. When the NHS was first founded in 1948, around 48% of people died before the age of 65 [5]. Today, that figure has declined to 12%, with the Office for National Statistics (ONS) estimating that by 2040 there will be over 17 million people in the UK aged 65 and above [6]. Osteoporosis is the most common age-related, chronic bone disease affecting the structural integrity of bones, predisposing them to fractures secondary to low-energy trauma, the so-called fragility fractures [7].

In recent literature, the concept of imminent fracture risk has been introduced and describes the risk of sustaining a fracture within the first two years following an initial fragility fracture [8]. Adequate treatment of patients following their initial fragility fracture has been shown to reduce subsequent risk of re-fracture by up to 50% [9]. Yet, despite the availability of a plethora of effective anti-osteoporosis therapies and an increasingly aging population, the proportion of patients receiving appropriate secondary prevention fracture care remains low.

In the UK, the outpatient management of patients with fragility fractures falls under the joint remit of general practitioners and fracture liaison services. Importantly, in some areas, fracture liaison services cannot prescribe medications and the onus often lies on the general practitioner to commence and monitor pharmacological therapy where appropriate. The results of this audit reveal that despite this joint care approach, patients are still being missed.

Recent guidelines from the NICE [3] recommend bisphosphonates as the first-line pharmacological agent for the secondary prevention of fragility fractures. Bisphosphonates are a class of anti-osteoporosis drugs that reduce osteoclast-mediated bone resorption by arresting the metabolic mevalonate pathway, resulting in loss of osteoclast function and eventual cell death by apoptosis [10]. Despite being the first-line agent in the secondary prevention of fragility fractures, these medications are not without their side effects, contraindications, and drug monitoring requirements. One relatively common side effect of this therapy is upper gastrointestinal irritation caused by a combination of local chemical gastritis as well as mucosal epithelial injury secondary to a reduction in the hydrophobic barrier of the upper gastrointestinal tract [11,12]. This adverse effect was reported by one patient in our study, who discontinued alendronic acid after a trial of therapy for a few months.

Low bone mass is almost always the etiology underlying increased bone fragility, and in the clinical setting, bone mass densitometry measurements using DEXA have been shown to demonstrate a strong predictive value on future fracture risk, with a single measurement being able to predict hip fracture risk for up to 25 years [13,14]. Four of the 10 patients included in the study had a DEXA scan completed following their index fracture. The FRAX score was calculated for the remaining patients and used in conjunction with national guidelines to inform decision-making regarding commencing pharmacotherapy.

Of the 10 patients found to be on neither bone protection nor vitamin D/calcium supplementation, one patient was previously trialed on a bisphosphonate, reported adverse effects, and declined a second-line agent. Two patients were noted to have contraindications to oral bisphosphonates (one patient had a history of esophagitis with benign strictures while the second had an unsafe swallow and was currently under the care of gastroenterology and speech and language therapy). They were both referred to the Older Person’s Rapid Access Clinic for zoledronic acid infusions. Using the FRAX tool, four patients met the criteria for measurement of their bone marrow densitometry and were subsequently referred for DEXA scans. One patient completed a five-year course of weekly alendronic acid following their initial fracture and was on a *drug holiday* [15]. One patient, with known chronic kidney disease and an estimated glomerular filtration rate of 34 mL/minute/1.73m^2^ was referred to the endocrinology team for advice regarding the commencement of denosumab, a second agent used in the treatment of osteoporotic bone disease [16]. Denosumab, is a monoclonal antibody that prevents the RANKL/RANK interaction, inhibiting osteoclast formation, function, and survival, thereby decreasing bone resorption [17]. The final patient was counseled and appropriately commenced on weekly alendronic acid supplementation.

In the UK, clear guidelines and clinical pathways exist for the management of patients following a fragility fracture. The introduction of the fracture liaison service bridges inpatient care to the community. General practitioners and primary care networks must appreciate the importance of early recognition of patients with fragility fractures and ensure that timely and appropriate bone-protecting therapies are instituted.

Limitations of this study include its retrospective design with the potential for selection bias. The sample size was relatively small (one primary care facility) and data were collected from electronic records, introducing the possibility of missing or incomplete records.

## Conclusions

The findings of this audit reveal that despite both general practitioners and fracture liaison services sharing responsibility for the outpatient care of patients with fragility fractures, patients are still being omitted, and opportunities to prevent future fractures are being missed. Local practices must conduct regular audits to ensure adherence to national standards. Following initial fragility fractures, patients should be proactively identified and evaluated to ensure that appropriate and timely treatment is commenced. These fractures are associated with significant morbidity and mortality with a pronounced impact on patients’ quality of life and the importance of preventing these fractures cannot be understated.

## Figures and Tables

**Figure 5:**
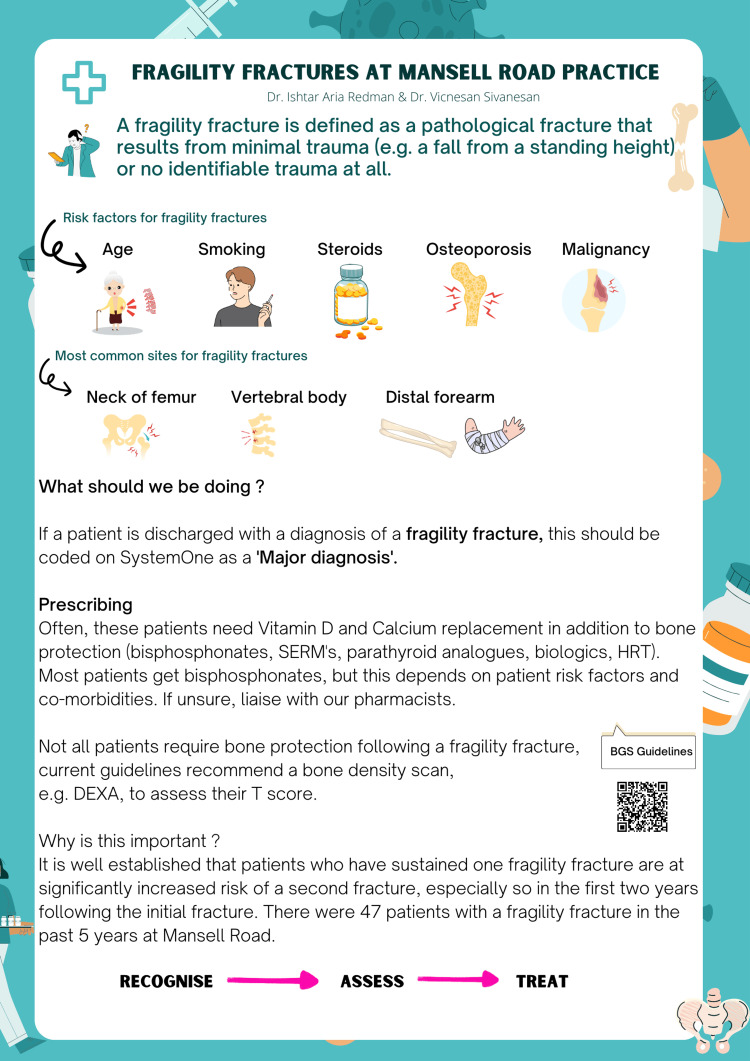
Poster created to improve knowledge of fragility fractures at the practice.
